# Early outcome of coronary endarterectomy combined with coronary artery bypass grafting among patients with coronary artery disease

**DOI:** 10.12669/pjms.42.(11AASC).15804

**Published:** 2026-04

**Authors:** Noor Us Sabahat, Syed Shahabuddin, Javeria Javed, Shahid Sami

**Affiliations:** 1 Dr. Noor Us Sabahat, MBBS. Department of Medicine, Aga Khan University Hospital, Karachi, Pakistan; 2 Dr. Syed Shahabuddin, MBBS, FCPS, FACS, FRCS. Department of Surgery, Aga Khan University Hospital, Karachi, Pakistan; 3 Dr. Javeria Javed, MBBS. Department of Surgery, Aga Khan University Hospital, Karachi, Pakistan; 4 Dr. Shahid Sami, FRCS. Department of Surgery, Aga Khan University Hospital, Karachi, Pakistan

**Keywords:** Coronary artery bypass, Cardiac surgery, Endarterectomy, Income Country, Lower-Middle- Surgical outcomes

## Abstract

**Background & Objective:**

Diffuse coronary artery disease (CAD) poses significant challenges for coronary artery bypass grafting (CABG), particularly when distal targets are heavily calcified. Coronary endarterectomy (CE) allows plaque removal and complete revascularisation, yet evidence on early outcomes remains limited in low- and middle-income countries (LMIC). This study aimed to evaluate 30-day outcomes of CE with CABG.

**Methodology:**

We conducted a retrospective review of adults who underwent isolated CABG with adjunctive CE at a high-volume tertiary centre between January 1995 and December 2021.

**Results:**

Eighty patients were included, with a mean age of 58 years and 90% were male. Mean body mass index (BMI) was 27.4, and mean ejection fraction (EF) was 48.5%. Median bypass time was 125 minutes and cross-clamp time 87 minutes. CE was most commonly performed on the left anterior descending artery (51%) and right coronary/posterior descending artery (41%). Left main disease >50% was present in 26.3%, and 91% had three-vessel disease. Recent myocardial infarction was noted in 22.5% within seven days and 36.3% beyond seven days. Most common early complications included prolonged ventilation (> 48 hours post-op) (12.5%), re-exploration (5%), and pneumonia (3.8%). About 17% required cardiac intensive care unit stay beyond 48 hours, and 53.8% received blood transfusion. Median hospital stay was six days. Operative mortality was defined as mortality during initial hospital stay and was 2.5%, with no strokes observed.

**Conclusions:**

Adjunctive CE during isolated CABG achieved acceptable early outcomes and low mortality in this LMIC setting. The findings support its role as a feasible strategy for diffuse CAD, warranting further prospective evaluation of long-term outcomes with propensity matched control group without CE.

## INTRODUCTION

Coronary artery disease (CAD) is the leading cause of mortality worldwide, responsible for nearly five million deaths each year in the developing world alone.^1^ The burden is particularly high in low and middle income countries (LMICs), where patients often present at a younger age with diffuse and calcified and obstructive atheromatous lesions.^2^ Limited access to percutaneous intervention further increases reliance on surgical revascularisation. Coronary artery bypass grafting (CABG) remains the standard of care for multivessel disease. However, diffuse CAD presents unique technical challenges when suitable distal targets for grafting are absent, necessitating adjunctive surgical strategies.

Coronary endarterectomy (CE), first described in 1957, is one such strategy.^3^ The technique involves removal of atheromatous plaque to restore the vessel lumen, thereby permitting bypass grafting.^4^ Early experience in the 1960s and 1970s was associated with high morbidity and mortality 3.6% vs none in a control group, which hindered its widespread adoption.^5^ With refinements in surgical technique, improved myocardial protection, and the use of dual antiplatelet therapy, contemporary outcomes have improved considerably.^4^,^6^

Recent evidence suggests that CE increases perioperative risk compared with CABG alone, though long-term survival appears comparable and hence justifying its use.^7^ However, the technique has its benefits as studies have shown that CE can accomplish revascularization in areas previously deemed ungraftable, with an added survival benefit.^4^ Meta-analyses support this trend, indicating acceptable durability in appropriately despite inherent bias selected patients.^7^ Long-term data from Shehada and colleagues demonstrate survival exceeding 75% at seven years, reinforcing the role of CE in complex disease.^8^ Nevertheless, most of these reports arise from high-income settings. Evidence from LMICs remains scarce, with only small retrospective series from Iran and Pakistan suggesting that CE combined with CABG can be performed safely with acceptable early outcomes.^9^,^10^

An important and underexplored subgroup includes patients with diffuse disease involving the left main coronary artery (LMD). Left main disease carries a high mortality risk when managed medically compared with surgical revascularization.^11^ It remains unclear whether the addition of CE to Left anterior descending artery (LAD) or other branches confers further perioperative risk in this already high-risk population.

Given these gaps, this study examines short-term outcomes of CE with CABG at a high-volume tertiary centre in an LMIC setting. We focus on perioperative complications, operative mortality, and the influence of LMD on early prognosis. These findings aim to inform surgical practice and guide risk stratification in comparable healthcare contexts.

## METHODOLOGY

A single centre retrospective observational study was conducted at the Aga Khan University Hospital in Karachi, Pakistan and included adult patients aged 18 years or older.

### Ethics approval:

It was obtained from the institutional Ethics Review Committee (ERC) with a waiver of individual informed consent granted under approval number ERC 2022-7132-20987; dated April 12, 20222.

### Inclusion & Exclusion Criteria:

Eligible participants were adults who underwent isolated CABG with adjunctive CE between January 1995 and December 2021. Patients who underwent concomitant procedures such as valve replacement or those with incomplete medical records were excluded.

Consecutive non-probability sampling was employed and medical record numbers of all eligible patients were obtained from the Health Information System (HIMS). The estimated sample size was 78 at a 95% confidence interval. During data extraction two additional eligible cases were identified which resulted in a final study population of 80 patients.

### Data collection:

Pre-operative variables (age, sex, BMI, smoking status, hypertension, diabetes mellitus, chronic kidney disease/dialysis dependence, cerebrovascular disease, history of MI< or >7 days ago, congestive heart failure, left ventricular ejection fraction) were extracted from patient charts. Three-vessel coronary disease was defined angiographically. Significant left main disease (LMD) was defined as ≥50 % stenosis and used for subgroup analyses.

Intra-operative data included operative urgency (elective vs urgent/emergent), cardiopulmonary bypass (CPB) time, cross-clamp time, number and type of grafts, and location of endarterectomised vessels.

### Outcomes:

The primary endpoints were operative mortality (death within 30 days of surgery or during the index admission) and major postoperative morbidity: peri-operative myocardial infarction (MI), defined as MI within the 24 hours preceding or succeeding the operative intervention; stroke; re-exploration for bleeding; deep sternal wound infection; leg wound infection; pneumonia and prolonged ventilation (>48 hour).

Secondary endpoints included CICU stay >48 h, transfusion of blood products and total hospital length of stay. All patients were followed until discharge or death; longer-term outcomes were not available.

### Statistical analysis:

Data were analysed using SPSS v.22. Continuous variables are presented as mean ± standard deviation or median (IQR) according to distribution; categorical variables were summarised as counts and percentages. Comparisons between patients with and without LMD were performed using the *t-*test, Mann–Whitney U or chi-square tests as appropriate. A two-sided *p* value <0.05 was considered statistically significant.

## RESULTS

**Fig.1:**
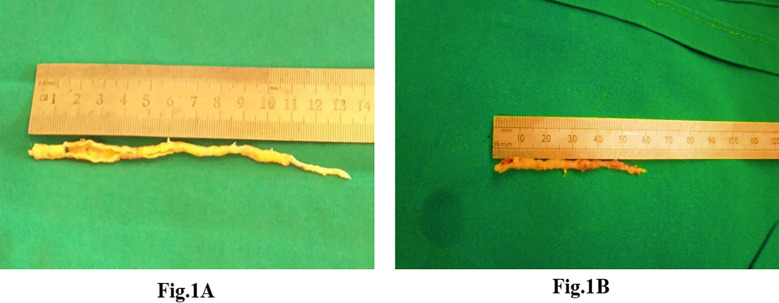
Atheromatous plaques extracted during coronary endarterectomy. **(A)** Left anterior descending artery (LAD) specimen. **(B)** Posterior descending artery (PDA) specimen.

A total of 80 patients underwent CE with CABG during the study period. The cohort was predominantly male, middle-aged, and carried a high burden of cardiovascular risk factors, including hypertension and diabetes. Nearly all patients had three-vessel disease, and over one-quarter had significant left main disease [Fig.1a and 1b]. Recent myocardial infarction was frequent, with one in five presenting within the preceding week. Baseline and operative details are summarized in Table-I.

**Table-I:** Baseline characteristics and operative details

Variable	Value
Age, years (mean ± SD)	58.0 ± 9.4
Male sex, n (%)	72 (90.0)
BMI, kg/m² (mean ± SD)	27.4 ± 6.0
Hypertension, n (%)	56 (70.0)
Diabetes mellitus, n (%)	54 (67.5)
Peripheral vascular disease, n (%)	7 (8.8)
Dialysis dependent, n (%)	1 (1.3)
Left ventricular ejection fraction, % (mean ± SD)	48.5 ± 15.0
Three-vessel disease, n (%)	73 (91.3)
Significant left main disease, n (%)	21 (26.3)
Recent MI < 7 days, n (%)	18 (22.5)
MI > 7 days, n (%)	29 (36.3)
Elective procedure, n (%)	74 (92.5)
On-pump CABG, n (%)	80 (100)
Site of CE	
• LAD, n (%)	41 (51.3)
• RCA/PDA, n (%)	33 (41.3)
• LCX, n (%)	4 (5.0)
Cardiopulmonary bypass time, min (median, IQR)	125 (105–150)
Cross-clamp time, min (median, IQR)	87 (70–107)
Number of grafts (median, range)	3 (2–4)

Operative mortality was 2.5% (two patients), and no perioperative strokes occurred. Prolonged ventilation was the most common complication, affecting 12.5% of patients. Pneumonia occurred in 3.8%, perioperative myocardial infarction in 5%, and re-exploration for bleeding in 6.3%. Major wound infections were rare, each affecting only one patient. More than half of the cohort required transfusion, and 17.5% remained in the cardiac intensive care unit for longer than 48 hours. Median length of hospital stay was six days.

### Left Main Disease Subgroup:

Patients were stratified into two groups: those with left main disease (LMD) and those without LMD. The differences in baseline clinical characteristics between both groups are summarized in Table-II. Cardiopulmonary bypass and cross-clamp times did not differ between groups.

**Table-II:** Baseline characteristics of patients in the LMD and non-LMD group.

Variable	LMD Group (n=34)	Non-LMD Group (n=46)	p-value
Number of patients	34	46	-
Median age (years)	61	60	0.42
Male gender (%)	83	88	0.51
Diabetes Mellitus (%)	65	54	0.22
Hypertension (%)	76	70	0.49
Chronic kidney disease (%)	18	7	0.09
Dialysis-dependent (%)	15	2	0.02
LVEF > 50% (%)	59	67	0.36

Postoperative morbidity was higher in the LMD subgroup. Prolonged ventilation occurred in 26.5% of LMD patients compared with 2.2% of non-LMD patients (p < 0.01). Pneumonia developed in 8.8% versus 0% (p = 0.03), and re-exploration was required in 11.8% versus 0% (p = 0.01). Operative mortality was higher in the LMD group (5.9% vs. 0%), although this did not reach statistical significance (p = 0.08). Transfusion requirement and CICU stay longer than 48 hours were more frequent in LMD patients but without significant differences. The information on carotid ultrasound was not collected during data abstraction; however, no perioperative strokes were observed in either subgroup.

## DISCUSSION

Our analysis of 80 patients undergoing coronary endarterectomy (CE) combined with coronary artery bypass grafting (CABG) demonstrates that this strategy can be performed safely in a low- and middle-income country (LMIC) setting. Despite advanced coronary disease and multiple comorbidities, operative mortality was 2.5% and no perioperative strokes occurred. Postoperative complications were manageable, and length of stay was acceptable. These findings suggest that CE, when applied selectively, represents a feasible revascularisation option for diffuse coronary disease in resource-constrained settings.

Large registry analyses and meta-analyses consistently report higher perioperative risk when CE is combined with CABG compared to CABG alone. Soylu et al. showed increased 30-day mortality, greater risk of perioperative myocardial infarction, and longer hospitalization.^12^ Similarly, Wang et al. demonstrated higher odds of perioperative complications across more than 60,000 patients, particularly in those with extensive LAD disease.^6^ More recent data from the Society of Thoracic Surgeons confirm increased operative mortality and myocardial infarction with CE, though survival curves converge with CABG alone after the first postoperative year.^13^

Against this backdrop, our results compare favorably. Mortality in our cohort was lower than most registry estimates, and stroke was not observed. These outcomes likely reflect careful patient selection, adherence to standardized techniques, and optimized perioperative care. They also highlight that, even in LMIC settings with constrained resources, outcomes can approximate those reported in high-income countries.

Published experience with CE from LMICs remains limited. A large Bangladeshi off-pump series of 1,000 patients demonstrated intensive care mortality below 2% and excellent early extubation times, reinforcing the safety of CE in carefully managed settings.^14^ Our study adds to this literature as one of the largest contemporary single-centre reports from South Asia. It underscores that CE, far from being a last-resort strategy, can be integrated into standard revascularisation practice when diffuse disease is common and alternatives are limited in a viable myocardial territory.

Although our follow-up was restricted to 30 days, existing literature supports the long-term durability of CE. Mid-term studies report graft patency rates between 70% and 80%, with superior outcomes when the left internal mammary artery (LIMA) is used for LAD reconstruction.^15^ Computed tomography angiography studies have documented patency rates approaching 90% under contemporary antiplatelet regimens.^16^ Survival rates around 75-80% and freedom from major adverse cardiac events above 60% at seven years have been described consistently.^8^ Importantly, differences in mortality between CE and CABG alone diminish after the first postoperative year.^6^,^13^ These findings support CE as a durable revascularisation strategy when technical principles are respected and antiplatelet therapy is optimized.

Approximately one-quarter of our patients had significant left main disease (LMD). Compared to those without LMD, these patients were older, more likely to have dialysis-dependent renal dysfunction, and experienced higher morbidity. These observations are consistent with published risk models in which LMD independently predicts adverse outcomes.^17^ They reinforce the importance of meticulous myocardial protection and vigilant postoperative monitoring in this subgroup. While our study was not powered to draw definitive conclusions, the findings suggest that LMD patients undergoing CE represent a particularly high-risk population who warrant special consideration in operative planning.

All patients in our series underwent on-pump CABG with CE. Literature comparing pump strategies is mixed. Some studies demonstrate no difference in early mortality or graft patency between on- and off-pump approaches.^6^,^15^ Off-pump CE may reduce costs, avoid cardiopulmonary bypass-related inflammation, and shorten ventilation time, which is particularly relevant in LMICs.^18^,^19^ Conversely, on-pump techniques provide a still and bloodless field, which is advantageous for extensive LAD or multivessel endarterectomy.^20^ Evidence therefore suggests that outcomes are driven more by patient selection and surgeon expertise than by pump strategy alone. Both techniques remain acceptable when tailored to operative circumstances and institutional resources.

In high-income countries, CE is often reserved for rare cases of diffuse disease. In contrast, it holds greater practical relevance in LMICs, where patients frequently present late, with calcified multivessel disease, and where percutaneous options are limited or unaffordable.^21^,^22^ Our experience shows that CE combined with CABG can be delivered safely and effectively in such contexts, offering complete revascularisation where otherwise limited options exist.

Several strategies can enhance outcomes in resource-constrained environments. Off-pump techniques, where feasible, may reduce costs and facilitate earlier extubation. Routine use of the LIMA for LAD grafting along with additional arterial grafts is essential for long-term patency. Dual antiplatelet therapy should be initiated early and maintained long-term. Selective use of CT angiography can aid in graft surveillance, though resource limitations may restrict universal application. Early extubation protocols, strict infection control, and rigorous glycaemic management help reduce morbidity. Finally, investment in surgical training and maintaining case volumes is crucial, as outcomes are closely tied to institutional and surgeon expertise.

### Strengths of the study:

This study makes a significant contribution to the limited literature on CE from LMICs. To our knowledge, it represents one of the largest single-centre series from Pakistan. Strengths include a well-defined cohort, standardized surgical approach, detailed perioperative outcomes, and subgroup analysis of patients with LMD.

### Limitations:

Several limitations must be acknowledged. The retrospective design introduces potential sources of bias, and the single-centre setting restricts the generalizability of our findings. The sample size was relatively small, which limited the power of subgroup analyses. This likely reflects missing data and occasional mislabelling of operative records over the extended study period. In some cases of diffuse coronary disease, multiple sequential grafts were used instead of endarterectomy, further reducing the number of eligible patients. The data abstraction concluded in 2021 hence the data from more recent years was not available to be included in the analysis. This study also lacks a control group due to which comparative analysis could not be performed. The left main disease subgroup was particularly small, which limits the strength of statistical comparisons. Follow-up was restricted to thirty days and therefore did not allow assessment of long-term patency or survival. Postoperative CT angiography was not performed routinely, which precluded direct evaluation of graft patency. Finally, data on long-term adherence to antiplatelet or lipid-lowering therapy were not available, although these factors are known to influence outcomes.

## CONCLUSIONS

Coronary endarterectomy combined with CABG can achieve acceptable short-term outcomes in LMIC settings, even among patients with diffuse and advanced coronary disease. Operative mortality and morbidity in our cohort were comparable to international benchmarks. Patients with LMD experienced higher complications, underscoring the need for careful myocardial protection, routine LIMA use, and vigilant postoperative management. While CE carries acknowledged perioperative risks, accumulating evidence supports its durability, with survival and graft patency approaching that of standard CABG in the long term.

Our study highlights the feasibility and utility of CE in resource-limited environments where diffuse disease is common, and revascularisation options are scarce. Future research should prioritize prospective registries, refined risk stratification models, standardized perioperative protocols, and multicentre collaborations to advance the safe and effective use of CE globally.

### Recommandations:

Future multicentre studies with longer follow-up and incorporation of imaging surveillance are needed to confirm and expand these findings.

### Authors contribution:

**NS:** protocol writing, data collection, analysis and writing and editing of the manuscript.

**JJ:**writing and editing of the final manuscript.

**SyS:** Conceptualization and supervision and final editing,

**ShS:** Supervision. Critical Review.

**SyS** is accountable for accuracy and integrity of the work.

All authors have reviewed and approved the final manuscript.

## Availability of data and materials:

The dataset analysed during the current study is not publicly available due to restrictions imposed by the Institutional Review Board but may be obtained from the corresponding author upon reasonable request.
